# Protein-Mediated Bimetallic Nanoclusters: Effect of Protein Nature on Structure, Optical Property and Cytotoxicity

**DOI:** 10.3390/nano16150909

**Published:** 2026-07-24

**Authors:** Bianka Torma, Gergely F. Samu, Gabriella Spengler, Edit Csapó, Árpád Turcsányi

**Affiliations:** 1 MTA-SZTE Lendület “Momentum” Noble Metal Nanostructures Research Group, Department of Physical Chemistry and Materials Science, University of Szeged, Rerrich B. Square 1, H-6720 Szeged, Hungary; torma.bianka@chem.u-szeged.hu; 2 Department of Molecular and Analytical Chemistry, University of Szeged, Dóm Square 7–8, H-6720 Szeged, Hungary; samugf@chem.u-szeged.hu; 3 Department of Medical Microbiology, Albert Szent-Györgyi Medical School, University of Szeged, Semmelweis utca 6, H-6725 Szeged, Hungary; spengler.gabriella@med.u-szeged.hu

**Keywords:** gold/silver nanoclusters, gamma-globulin, lysozyme, serum proteins, structure analysis, cytotoxicity

## Abstract

Bimetallic nanoclusters (NCs) containing gold and silver were prepared by template-assisted synthesis using human serum albumin (HSA) via a newly optimized fabrication route at 25 °C. Additionally, following this procedure, we also reproducibly synthesized further Au/Ag NCs, containing a similar Au:Ag ratio, using bovine serum albumin (BSA), lysozyme (LYZ), transferrin (Tf), and gamma-globulin (γG). The aim was to highlight the importance of experimental conditions of the synthesis (e.g., metal ion: protein molar ratio and metal and protein concentrations, as well as synthesis time, temperature, and pH) for the composition, structure, and optical features of the protein-stabilized ultra-small-sized products. Circular dichroism (CD) spectroscopy revealed that the partial unfolding of the stabilizing proteins is primarily caused by the alkaline synthesis environment rather than the nanocluster formation itself. Furthermore, X-ray photoelectron spectroscopy (XPS) and inductively coupled plasma mass spectrometry (ICP-MS) successfully confirmed the presence of mainly metallic (Au^0^) core structures alongside Ag^0^/Ag^+^ species, providing the actual metal-to-protein ratios after purification. As a new result, cytotoxicity of these bimetallic NCs was determined by using doxorubicin-sensitive Colo205 and CCD-19Lu human normal fibroblast cell lines, and their antibacterial activity was also evaluated using four different Gram-positive and Gram-negative bacterial strains.

## 1. Introduction

The transition from traditional monometallic nanoparticles (NPs) to ultra-small, atom-precisely engineered noble metal nanoclusters (NCs) has redefined the landscape of nanophotonics and biotheranostics [1,2]. Among these, protein-stabilized bimetallic gold/silver nanoclusters (Au/Ag NCs) have emerged as particularly promising candidates in these fields. They often surpass their monometallic counterparts in stability, biocompatibility, and optical performance [3,4]; therefore, these NCs can become forerunners in molecular sensing and medical imaging [4,5,6,7]. By utilizing globular proteins—such as bovine serum albumin (BSA), human serum albumin (HSA), lysozyme (LYZ), transferrin (Tf), and gamma-globulin (γG)—as “bioreactors” and stabilizing templates, researchers can achieve a level of structural control and functional synergy hardly attainable with simple synthetic ligands [2]. The choice of protein is critical, as the unique amino acid sequence and folding architecture control the nucleation and growth of the metal core; furthermore, additional functionality can be preserved from the native protein. Serum albumins (BSA and HSA) serve as reference heartland templates due to their well-defined hydrophobic pockets and abundant cysteine/tyrosine residues capable of directing cluster growth. Including both HSA and BSA allows for a direct comparative assessment of how subtle species-specific sequence variations and binding microenvironments influence core formation and photophysical dynamics under identical alkaline conditions [8]. Lysozyme (LYZ) was selected to represent a compact, low-molecular-weight (~14.4 kDa) globular protein with a high isoelectric point (pI ≈ 11), providing a distinctly different electrostatically driven microenvironment for metal precursor interaction compared to the acidic serum albumins. Transferrin (Tf) and gamma-globulin (γG) were incorporated into the comparative matrix to evaluate larger biomolecules (>70–150 kDa) with intrinsic physiological recognition properties. Tf possesses high-affinity metal-binding domains, whereas γG represents immunoproteins rich in β-sheet architectures. Demonstrating the successful, reproducible synthesis of stable NCs within these functional carriers is pivotal for developing targeted biotheranostic nanoprobes that retain structural integrity and low cytotoxicity [9]. The cytotoxicity of protein-stabilized Au and Ag NCs is remarkably low compared to their chemically synthesized counterparts, primarily due to the biocompatible protein corona that shields the metal core. The optical characteristics of NCs differ greatly from plasmonic NPs; these nanostructures possess discrete electronic states due to their molecule-like composition. The number of building atoms, particle shape, and other properties define their distinct electronic transitions in the metal cores [10,11], providing great optical tunability for different applications, albeit with usually very low quantum yields (QY) that are expected [12]. Long photoluminescence lifetimes characteristic of protein-stabilized metallic NCs can be utilized in applications such as time-resolved imaging, with emissions in the red-near-infrared region enabling better tissue penetration. The primary advantage of bimetallic NCs over monometallic derivatives lies in the tunability of their optical properties [13]. For QY, the bimetallic NCs often exhibit significantly higher luminescence than pure Au NCs, leveraging the superior radiative rates of silver while maintaining the chemical robustness of gold [14]. Moreover, these alloy materials demonstrate complex photophysical dynamics, with lifetimes spanning from nanoseconds to microseconds [5,14]. This diversity is essential for time-resolved fluorescence imaging, allowing for the effective suppression of background autofluorescence in biological tissues. By adjusting the Au/Ag stoichiometric ratio, the emission can be precisely tuned across the visible (Vis) to the near-infrared (NIR) spectrum [14], optimizing them for deep-tissue penetration. In this article, we conduct a comprehensive analysis in which we compare the role of the experimental conditions of optimized production protocols in the formation, metal core composition, protein structure, and optical features of different bimetallic Au/Ag NCs stabilized by various proteins (BSA, HSA, LYZ, γG, and Tf). Building upon our preliminary synthetic protocols for individual protein systems [15,16,17], this work establishes a unified, systematic benchmarking framework. By applying standardized optimization strategies across this diverse set of protein templates (differing in molecular weight, secondary structure ratios, disulfide linkages, and surface charge density), we aim to directly correlate the physical-chemical traits of the protein matrix with the resulting core compositions, XPS data for oxidation state, optical quantum yields, and biocompatibility profiles. In the case of HSA, for better comparability, we produced the NCs via a newly optimized production process; thus, the characterization of the HSA-Au/Ag NCs is presented as a new result in the article.

## 2. Materials and Methods

### 2.1. Materials

Serum albumin from bovine blood (BSA, ≥98%, 66 kDa, heat shock fraction, protease, fatty acid and essentially globulin free), lysozyme (LYZ, ≥90%, 14,388 Da, from chicken egg white), gamma-globulin from bovine blood (γG, >99%, agarose gel electrophoresis), human apo-transferrin (Tf, BioReagent, 98%, 79,570 Da, agarose gel electrophoresis, suitable for cell culture), albumin from human serum (HSA, ≥96%, 66.5 kDa, agarose gel electrophoresis) were supported by Sigma Aldrich (Budapest, Hungary). Hydrogen tetrachloroaurate (HAuCl_4_ × 3H_2_O, 99.9%, Au content 49%) was purchased from Alfa Aesar (Ward Hill, MA, USA), while silver nitrate (AgNO_3_, a.r. grade, 99.9%), sodium hydroxide (NaOH, 98%), sodium dihydrogen monophosphate (NaH_2_PO_4_ × 2H_2_O, 99%), disodium hydrogen monophosphate (Na_2_HPO_4_ × 12H_2_O, 99%), sodium chloride (NaCl, 99%) were from Molar Chemicals (Halásztelek, Hungary). For fluorescence quenching studies, potassium iodide (KI, puriss) from Molar Chemicals and acrylamide (≥99%, suitable for electrophoresis) from Sigma Aldrich were used. Hydrogen chloride solution (37% solution) was obtained from VWR (Debrecen, Hungary). All substances used in this work were of analytical grade, and no further purification was made. For all solutions, ultrapure water was used from a Millipore system (18.2 MΩ cm at 25 °C) (Darmstadt, Germany).

### 2.2. Preparation of Protein-Stabilized Bimetallic Gold/Silver NCs

To prepare bimetallic gold/silver NCs stabilized by different proteins (namely BSA [15], LYZ [15], Tf [16], γG [17]) exclusively in an aqueous medium, our previously optimized and published protocols were used [15,16,17]. The number of replicated samples was n = 3 in each set of measurements. For the investigations regarding biocompatibility, monometallic Au NCs were also prepared based on [15,16,17]. In the case of HSA, we developed a new production route, slightly modifying a previously published method [18], following the route already established for the aforementioned proteins. In this case, 45 mg of the HSA protein was dissolved in 5 mL of ultrapure water for 15 min, then the metal precursor salt solutions were mixed in a 9:1 molar ratio for Au and Ag. In detail, 345 µL of 10.0 mM HAuCl_4_ and 37.5 µL of 10.0 mM AgNO_3_ solutions were added to the protein-containing sample, and we supplemented the sample volume with 127.5 µL of water to reach a 5.51 mL final sample volume (similar to previous preparation procedures). The resulting solution was stirred for 5 min to allow bonding between the protein and the metal ions. After this step, 370 µL of 1 M NaOH solution was added to set a high pH (pH > 12) for metal reduction. At such a high pH, the tyrosine residues of HSA are capable of reducing Au- and Ag-ions to their zero-valent states, and the protein organizes the growth into NCs, also hindering superficial growth into plasmonic metal NPs. This step was conducted without stirring, protecting the NC samples from ambient light. The reduction took place at 25 °C for 24 h. The resulting NCs were pale yellow in color and showed intense orange-red emission under UV light (λ_ex_ = 365 nm). The NCs were dialyzed using a semi-permeable cellulose-based membrane, which had a molecular weight (M_W_) cut-off value of 14 kDa, which was sufficiently high to prevent the leakage of the protein (M_W_ = 66.5 kDa) and the NCs. Cleaning was done in two steps, where the sample volume (5.88 mL) was first put into 1 L of ultrapure water for 90 min, then the dialyzing medium was switched to another batch of fresh water, and the second step took 60 min. The resulting sample showed no changes in the shape of the emission spectrum, although the intensity became somewhat lower due to the loss of the metal (see the results for metal content in Section 3.4).

### 2.3. Determination of the Optical Properties

The optical properties of the synthesized NCs (absorbance and fluorescence) were determined using a Jasco V770 spectrophotometer and a Jasco FP-8500 spectrofluorometer (ABL&E-JASCO Magyarország Kft, Budapest, Hungary) using n = 3 parallel samples. Absorbance spectra were recorded in the 200–800 nm region using 1 nm bandwidth and 1 nm data interval, while fluorometric data were collected in the 340–700 nm region for HSA (λ_ex_ = 325 nm), 380–700 nm region for BSA, γG, Tf (λ_ex_ = 365 nm) and 385–700 nm for LYZ-based derivatives (λ_ex_ = 370 nm), respectively. The bandwidth at the excitation and emission regions of the device were both kept at 2.5 nm (in the case of synthesis time optimization, bandwidth values were raised to 5 nm; data interval was 1 nm, and response time was 50 ms). A Jobin Yvon FluoroMax-4 (Horiba, Retsch Technology GmbH, Haan, Germany) spectrofluorometer was used to assess iodide and acrylamide quenching data (4 nm bandwidth, 1 nm data interval, 0.1 s response time).

### 2.4. Fluorescence Lifetime and QY Measurements

In order to assess the optical properties of NCs better, fluorescence lifetime data were collected at different wavelengths (for protein signals, 460 nm was used; for NC signals, 640 nm for BSA-, HSA-, 620 nm for LYZ I-, 580 nm for LYZ II-, 660 nm for γG- and Tf-Au/Ag NCs were used, respectively), using a Horiba DeltaFlex time-correlated single-photon counting (TCSPC) device. The applied light source was a pulse-operated laser with an emission wavelength of 371 nm. The repetition rate used for the measurements varied between 50 kHz and 8 MHz (with the corresponding time ranges of 13 µs and 100 ns). Slit values were used in the range of 6–16 nm. The impulse response functions (IRFs) were collected with every measurement setting. The measurement data were analyzed using the built-in software of the device (EzTime), using estimations based on the following sum equation (Equation (1))

(1) N=a+∑i=1nbi×e−tτi

where *N* is the number of counted photons, *a* is a constant, *b_i_* is the i-th pre-exponential, *t* is the elapsed time (in ns or µs) and *τ_i_* is the i-th lifetime component. The value of n was 3 for measurements at the higher wavelength peak (580–660 nm) and 4 for the protein peak (460 nm). For QY measurements the Jasco FP-8500 spectrofluorometer was equipped with an integrating sphere (ILF-835) as sample holder (diameter is 100 mm). The measurements were executed as in our previous work [16]. For the calculations, calibration spectra were recorded using a Jasco Calibrated Light Source-WI (ESC-842) (ABL&E-JASCO Magyarország Kft, Budapest, Hungary), and were used subsequently for spectral correction. The calculation of QY values was done using the ABL&E Jasco SpectraManager 2.0 program, with the number of replicate measurements being n = 3.

### 2.5. Composition and Structure Determination

In order to determine the actual metal concentration and composition of the bimetallic NCs after purification, inductively coupled plasma mass spectrometry (ICP-MS) measurements were carried out. The system consisted of a quadrupole Agilent 7700X instrument, an Agilent I-AS type autosampler, a MicroMist microflow concentric pneumatic nebulizer, and a Scott-type spray chamber with Peltier cooling (Agilent, Santa Clara, CA, USA). The concentrations of the metals in the samples were determined by dissolving the NCs in hot aqua regia for 1 h. For gold, the calibration was carried out using the Agilent Multi-Element Calibration Standard-3 (Agilent, Santa Clara, CA, USA), and for silver, the Inorganic Ventures IV-ICPMS-71A. The final values were recorded at 0–100 µg L^−1^ concentrations using three parallel measurements, obtaining an RSD value of 0–6%. To determine the effect of NC formation on the secondary structure of the proteins, circular dichroism (CD) measurements were conducted using an ABL&E-Jasco J-1100 CD spectrometer (ABL&E-JASCO Magyarország Kft, Budapest, Hungary). The spectra were recorded in the 190–400 nm region by averaging three parallel measurements on each sample, using a 5 L min^−1^ flow rate of N_2_ to purge oxygen and ozone from the measurement chamber. In each case, samples using only the protein were made to check whether the changes in protein structure were caused by the NC formation or just by the high pH during the reaction. For these samples, the volume of metal salt solutions was simply changed to water while retaining every other step (for further information see references [15,16,17]). Samples containing the proteins or the NCs were investigated in 10 mM Na_2_HPO_4_ solution (abbreviated as P). As the medium increased the high-tension voltage (HT) of the device, unreliable readings were gained at lower wavelengths; therefore, these regions were cut from the spectra used for evaluation. To determine the oxidation state of the gold and silver metal atoms in the clusters, X-ray photoelectron spectroscopy (XPS) measurements were performed with a SPECS instrument equipped with a PHOIBOS 150 MCD 9 hemispherical analyzer (SPECS Surface Nano Analysis GmbH, Berlin, Germany). For this measurement, the lyophilized powders of the NC samples with higher metal content (0.94–0.97 mg of metal, except for Tf-Au/Ag NCs, where 0.30 mg metal was used) and lower protein amounts (15 mg of BSA, HSA and γG, 20 mg of LYZ and 3 mg of Tf) were prepared (see Table 1 for exact compositions). This was necessary because the original NCs had low metal content, and the presence of proteins bound to the metal significantly attenuated the signal. The analyzer was operated in fixed transmission mode with 40 eV pass energy for acquiring survey scans. For the high-resolution scans, 20 eV pass energy was used. Al K_α_ radiation (hν = 1486.6 eV) was used as an excitation source and operated at 150 W power. Twenty scans were averaged to obtain a single high-resolution spectrum. In all cases, an electron flood gun was used for charge compensation. Charge referencing was performed for the adventitious carbon C 1s peak (285.0 eV). For spectrum evaluation, CasaXPS commercial software package was used.

### 2.6. Cytotoxicity Studies

Cell lines and different culture conditions for cytotoxicity experiments were as follows: in the case of Colo205, the doxorubicin-sensitive cell line (ATCC^®^CCL-222^TM^) was obtained from LGC Promochem, Teddington, UK. The cells were cultured in RPMI-1640 supplemented with 10% heat-inactivated fetal bovine serum (FBS), 2 mM L-glutamine, 1 mM sodium pyruvate, 10 mM HEPES, nystatin (100 U/L), and a penicillin–streptomycin mixture (10 mg/L). The cell lines were incubated at 37 °C in a 5% CO_2_–95% air atmosphere. On the other hand, the CCD-19Lu human normal fibroblast cell lines (ATCC^®^CCL-210^TM^) were purchased from the American Type Culture Collection (ATCC, Manassas, VA, USA). The cells were cultured in Eagle’s Minimal Essential Medium (EMEM) supplemented the same way as the Colo205 cell line. The cells were also incubated under identical conditions. The cells used for the cytotoxicity measurements were first detached using a 0.25% Trypsin-Versene (EDTA) solution for 5 min at 37 °C. The adherent CCD-19Lu cells were seeded in 96-well flat-bottomed microtiter plates for 24 h before the assay. This step was omitted for the semi-adherent Colo205 cells. For both cell lines, the cell number was 1 × 10^4^ cells/well in 100 µL culture medium. The stock solutions of the tested samples (namely LYZ I-, γG-, Tf- and HSA-Au/Ag NCs, and also Au NCs) were diluted sequentially in the culture medium in the microtiter plates, performing three parallel sets of measurements. As a reference drug, doxorubicin (DOX) was used. In the case of CCD-19Lu cells, the NC samples were diluted in a separate plate and then transferred to the plates containing the adherent CCD-19Lu cells. The final volume for each well was 200 µL for both cell lines. The culture plates were incubated at 37 °C for 72 h, and at the end of the incubation period, 20 µL of MTT solution (thiazolyl blue tetrazolium bromide dissolved in PBS at a concentration of 5 mg∙mL^−1^) was added to each well. After another incubation at 37 °C for 4 h, 100 µL of sodium dodecyl sulfate solution (SDS, 10% dissolved in 0.01 M HCl) was added to every well, and the plates were incubated again at 37 °C overnight. Cell growth was determined by measuring the optical density (OD) at λ = 550 nm (reference at λ = 630 nm) with a Multiscan EX ELISA reader (Thermo Labsystems, Cheshire, WA, USA). The IC_50_ values were calculated on GraphPad Prism software (version 5.00 for Windows; GraphPad Software, San Diego, CA, USA) by using a nonlinear regression curve fit. Cell viability was calculated as a percentage relative to the untreated control cells (incubated in culture medium only), which was considered 100% viable. All cytotoxicity tests were performed in triplicate (technical replicates) and repeated in at least three independent biological replicates (n = 3).

### 2.7. Antibacterial Activity Studies

The following bacterial strains were chosen: Gram-positive *Staphylococcus aureus* (American Type Culture Collection—ATCC–25923) as a methicillin-susceptible reference strain; methicillin-resistant *S. aureus* (MRSA, ATCC 43300); Gram-negative *Escherichia coli* (ATCC 25922) and *Klebsiella quasipneumoniae* (ATCC 700603). Based on the Clinical Laboratory Standard Institute (CLSI) guidelines, the Minimal Inhibitory Concentration (MIC) values of the observed samples were determined in 96-well plates. The stock solutions of the different samples were diluted in 100 µL of Mueller Hinton Broth, then 10^−4^ dilutions of overnight bacterial culture in 100 µL of the medium were added to each well, with the exception of the medium control wells. The plates were then incubated at 37 °C for 18 h. At the end of the incubation period, the MIC values of the tested NCs were determined by visual inspection.

## 3. Results and Discussion

### 3.1. Synthesis Optimization of HSA-Au/Ag NCs

**Figure 1:**
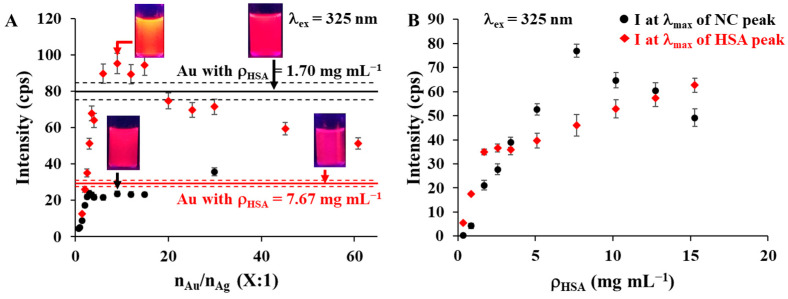
(**A**) Fluorescence intensities of several samples as a function of n_Au_/n_Ag_ ratios (●: *ρ*_HSA_ = 1.70 mg∙mL^−1^ ♦: 7.67 mg∙mL^−1^) with two representative photos of the NC samples (at 9:1 n_Au_/n_Ag_ molar ratio) under UV light. The lines represent the emission intensities of pure Au NCs made with the appropriate *ρ*_HSA_ values with the photos of the NC samples under UV light. (**B**) Fluorescence intensities of several samples as a function of *ρ*_HSA_ using 9:1 n_Au_/n_Ag_ molar ratio. The λ_ex_ is 325 nm.

During the preparation of HSA-Au/Ag NCs, different parameters were changed in a wide spectrum to observe their effect on NC formation and fluorescence. The aim of the optimization was to reach the highest fluorescence intensity by changing the investigated parameter, while keeping the others intact. The original method of NC synthesis was derived from the preparation of HSA-Au NCs [18]. In this case 4 mg protein was dissolved in 2 mL ultrapure water, then 204 µL HAuCl_4_ solution (10.0 mM) was added (0.40 mg total Au content). The formation of NCs was initiated by adding 148 µL 1 M NaOH solution to reach a high enough pH value for tyrosine residues in HSA (final protein concentration was 1.70 mg∙mL^−1^). In these conditions, Tyr residues are able to reduce metal salts (especially Au) into their zero-valent state [19], and the formation of the small NCs is endorsed by the protein molecule, which coordinates and also restricts the growth of the very small particles. Finally, we obtain samples with a pale pink color and a distinct red emission at ~635 nm (I ~ 80 cps, presented with a black line). For bimetallic NCs—including gold and silver—the molar ratio of the metal precursors was changed in the region of Au:Ag 1:0–0:1. Introducing silver into the NC cores usually comes with great changes in NC emission and color. In this case, at very small amounts of Ag, the emission intensity quickly dropped (at 29.9:1.0 n_Au_:n_Ag_ ratios, a ~55% decrease occurred, see Figure 1A, marked with ●) and λ_em, max_ did not change significantly (from 635 nm to 643 nm at 9:1 n_Au_/n_Ag_ composition).

Further increase in Ag content did not heavily change intensity readings but greatly changed the position of the peak (from 643 nm to 666 nm—6:1 n_Au_/n_Ag_—, then to 652 nm at 3:1 n_Au_/n_Ag_ composition). The changes observed in this set of experiments were similar to our studies with γG [17], albeit uncommon for the similar BSA protein [15]. In the next procedure we gradually changed the protein concentration in each sample, from 0.34 mg mL^−1^ to 15.29 mg∙mL^−1^ (Figure 1B).

At very low concentrations (0.34 mg∙mL^−1^), only larger NPs could be obtained, as the amount of HSA was too low to stabilize the small NCs and to prevent the growth of smaller clusters into larger NPs. At intermediate levels of HSA (0.85 mg∙mL^−1^), the main product was still NPs; however, a small amount of NCs was also formed, as the emission spectrum showed their distinctive orange-red emission peak. At 1.70 mg∙mL^−1^, the full amount of metal changed into NCs, and by increasing the concentration of HSA, higher intensities could be reached, topping at 7.67 mg∙mL^−1^. At high protein concentrations, the λ_em,max_ was around 606–609 nm, quite different from the observed 643 nm in the first experiment (*ρ*_HSA_ = 1.70 mg∙mL^−1^). Based on this, we repeated the first set of experiments focusing on n_Au_/n_Ag_ ratios but using the elevated *ρ*_HSA_ = 7.67 mg∙mL^−1^ (Figure 1A, marked with ♦). In this series, Au NCs showed lower emission intensity (I ~ 30 cps, presented with a red line) centered at λ_em,max_ = 654 nm, but even very small amounts of silver greatly changed the optical properties of the final product. The color quickly changes from pale pink (Au NCs) to pale yellow (Au/Ag NCs) at a 1:30 molar ratio, and the emission maximum shifts from 654 nm to 611–619 nm. This shift actually happens in two steps: first, the emission characteristic of Au NCs (654 nm) changes to 587 nm (two overlapping peaks can be seen). At larger Ag content, the emission gradually shifts to 610–620 nm, reaching maximum emission intensity (I ~ 95 cps) at 9:1 n_Au_/n_Ag_ molar ratio (this effect is discussed more in Section 3.2). A further increase in Ag content quickly reduces intensity but does not change the position of the peak. Moving on to temperature control, several articles pointed out its importance during NC synthesis [20,21,22]. In our case, the preparation of NCs was conducted at six different temperatures: 10, 17, 25, 32, 40 (original), and 60 °C (presented in Figure S1A). Based on the results, the synthesis at room temperature (25 °C) provides the highest emission intensity, with a mild blue shift in emission. The last parameter we investigated was metal concentration. In this case, the optimization was aimed at the highest normalized fluorescence intensity (the intensity value divided by the total metal concentration in mM). We observed the 0.13–1.30 mg∙mL^−1^ region and found that 0.33 mg∙mL^−1^ (75% of original) gave the highest emission (Figure S1B). Synthesis time dependence was evaluated on a sample with optimized parameters and in undiluted form using λ_ex_ = 365 nm. Based on our measurements, NCs reach optimal intensity in 24 h (the first twelve hours of synthesis are presented in Figure S2A) and maintain it for 4 h within statistical error, then intensities start to decline. As Figure S2B showed, NC emission starts building slowly (centered around 570 nm), then a fast increase is measured, with a red-shift to 610–615 nm.

### 3.2. Evaluation of NC Synthesis Parameters

**Figure 2:**
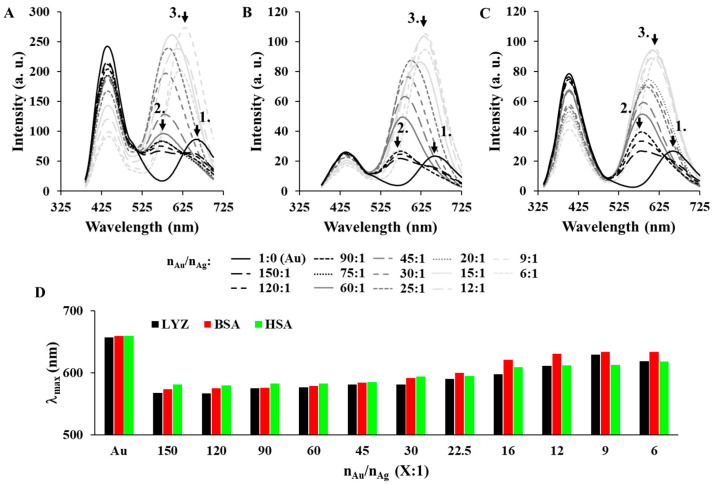
The effect of silver content on the emission profiles of (**A**) LYZ- (**B**) BSA- and (**C**) HSA-stabilized Au/Ag NCs, noting three major contributors to total emission with arrows 1–3. (**D**) The position of NC emission (λ_max_) at different n_Au_/n_Ag_ molar ratios.

In this section we intend to give a general summary on bimetallic Au/Ag NC formation using protein templates. During our previous works, several considerations on the determining parameters were assembled. The order of discussed synthesis parameters is the same as in Section 3.1. We investigated the following proteins: BSA, LYZ, γG, Tf and HSA. These are biomolecules with varied size, composition, and structure; thus, they have very different roles in organisms and can be used in many fields. Based on these differences, the formation of NCs would be expected to be quite different with every protein, except BSA and HSA, which possess very similar structures and functions. In spite of their similarity, we know that they have different binding sites and slightly different secondary and ternary structures, so the NCs formed with these two molecules are expected to be similar. The effect of the gold:silver molar ratio is the most determinative of the optical properties. Pure Au NCs usually show an emission peak in the red region, around 640–660 nm. This emission usually comes with a unique excitation peak centered around 500 nm [8]. The Au NCs also have a distinct pink color, although their absorbance was too low to be visible on the spectrum compared to the absorbance of the protein (see Figure S3A–C). Furthermore, as NCs are small dispersed particles, the light scattering also complicates the determination of their absorbance profile. Introducing silver into the structure of the NCs greatly changes the optical properties. In the finalized constitution, the emission maximum shifts to larger energies, usually 600–630 nm, and the emission intensity is usually higher than the value of Au NCs (the only exception was γG). The shift in emission maximum is also accompanied by a shift in excitation: the peak corresponding to the NCs shifts lower, overlapping with the protein excitation at 440 nm (see Figure S3D–F, where a NC with an intermediate composition of n_Au_/n_Ag_ = 45:1 was tested). This is also visible by the naked eye, as bimetallic NCs are yellow. A basic assumption of the process would be that slowly increasing the amount of silver would continuously change the emission wavelength and intensity, reaching peak value at a certain composition (e.g., n_Au_/n_Ag_ = 9:1 for BSA-stabilized NCs), then decreasing emission intensities would be seen. As we found out, this is not the case with LYZ [15], BSA and HSA (see Figure 2A–C; γG and Tf were not investigated).

In the case of LYZ, two metal compositions gave high intensities, at n_Au_/n_Ag_ = 6:1 with orange emission (620 nm) and n_Au_/n_Ag_ = 16:1 (600 nm). We investigated this region more thoroughly in a previous article [15], and found that even very low amounts of silver can change the emission. Using n_Au_/n_Ag_ = 150:1 composition, two emission peaks can be found, one at 660 nm (Au NCs) and another at 570 nm, which can be connected to newly formed bimetallic NCs. This change is also visible in color, as the pink color gradually turned to yellow. If we further increase the silver content, all of the Au NCs disappear, and a new emission peak appears centered around 600–610 nm. This new peak can be appointed to another bimetallic NC composition, but with a higher silver content. This phenomenon could also be determined for BSA and HSA (Figure 2B,C), with somewhat different n_Au_/n_Ag_ ratios, but with the same emission maxima (see Figure 2D).

Protein concentration also has an effect on NC emission. For Au NCs, usually lower concentrations (1.7–3.4 mg∙mL^−1^) give optimal emission rates, and below 1.7 mg∙mL^−1^ the NC formation is not supported by the protein. In these cases, the protein is unable to properly restrict the growth of the metal cores, and instead plasmonic NPs are formed, as is clear from the color and the lack of fluorescence. In the case of bimetallic Au/Ag NCs, this restriction also applies, but the optimal emission could be reached at much higher concentrations, usually around 7.7–12.7 mg∙mL^−1^. Very high protein levels cannot elevate the intensity further; instead, they decrease it, and can also cause colloidal stability issues, e.g., aggregation or even gel formation [15]. Synthesis temperature proved to be a determining factor in optical properties. Generally higher values (T > 40 °C) caused the emission intensity to be lower, and a red-shift could be detected. In turn, lower temperatures (T = 10–32 °C) give higher intensities with a blue shift, although the formation of the NCs becomes slower. The optimal temperature value was found to be 25 °C for every protein. While higher temperatures are usually beneficial for NC syntheses, especially with small ligands [20,21], proteins are sensitive to heat; therefore, temperatures over 40 °C cause denaturation, decreasing emission intensity and colloidal stability [15,17]. Total metal concentration can give rise to the question: what is considered high intensity in emissions for NCs? In our works [15,16,17] we usually considered the emission based on metal concentration (in units of cps/mM metal). This decision is also flawed, as NCs are composed of many metal atoms, usually in many conformations, which can also influence their properties. Furthermore, the presence of two metals in the NC cores means that different n_Au_/n_Ag_ compositions can be expected in one sample, with some kind of distribution (for example, a 9:1 distribution does not mean that all NCs have this composition). Aside from this, we found that usually lower metal contents (0.16–0.33 mM) are better to reach higher intensities. Synthesis time is also an important value to control for optimal intensity. While NCs with small ligands (e.g., amino acids) can take 1–3 days [21,23] or higher [24] to reach ideal fluorescence emission, NCs with proteins need at most 24 h, or much less with assisting tools, such as microwave irradiation [5]. In our case, synthesis times as low as 16 h can be enough to reach high enough emissions for work [15,17], without using any external help. Nevertheless, stable emission intensities were obtained only after 16–24 h.

### 3.3. Comparison of the Optical Properties

Fluorescence excitation and emission spectra are very important to NC characterization. In the case of our NCs, these spectra provided an insight into their optical properties. Based on the results published previously [16], we found that excitation spectra are very similar to the individual protein excitation spectra, while new peaks related to the NCs also emerged. Looking at the emission, we can see that beside the protein peak centered around 420–460 nm, the peak related to the NC appears between 566 and 633 nm for the bimetallic species. The position of this peak heavily depends on the Au/Ag ratio, but protein type and concentration and synthesis temperature can affect this aspect. Note that the excitation wavelength used to achieve maximal emission intensity heavily depends on the concentration, especially that of the protein. High-concentration NC samples (BSA, LYZ I, and LYZ II) show the excitation maximum at 365–370 nm, while for more dilute ones, the maximum appears at 313–321 nm (HSA and γG), or even as low as 275 nm (Tf). At very high dilutions (100×), the absorbance at lower wavelengths decreases into the measurable region; as such, excitation light is not completely absorbed by the sample. This effect is clearly visible on absorbance spectra of different concentrations. This measurement was conducted on the HSA-Au NCs and Au/Ag NCs (with n_Au_/n_Ag_ = 45:1), and the results are shown in Figure S3. From the spectra we can see that the excitation peak centered around 233 nm is completely quenched by self-absorption in undiluted NC samples, and emitted light is also absorbed. What is important to note is that there are some peaks appearing on the excitation spectrum (and are barely visible on the absorbance spectrum) that belong specifically to the NCs. The most important one is the excitation peak centered at 500 nm for Au NCs and 440 nm for Au/Ag NCs. This peak clearly belongs to the main NC emission at 660 or 570 nm [8]. Another peak of interest appears at λ_ex_ = 360–370 nm. The appearance of the corresponding emission peak can be detected around 440 nm and remains at every larger wavelength; thus, it contributes to NC emission (see the excitation spectra in Figure S3). Lifetime measurements conducted on every investigated NC showed that emission processes can be grouped into two major groups: the first group contains the protein-related emissions (centered at λ_em_ = 460 nm), which have very similar lifetimes regardless of the protein. As summarized in Table 2, usual lifetime components are 0.185–0.311 ns, 0.99–1.60 ns, 3.25–4.68 ns and 9.31–13.4 ns.

These emissions can be connected to tryptophan residues, as the first small values are purely from Trp and the third is from the Trp residue in a lipophilic environment [25]. The fourth component could not be connected to Trp; this value should belong to one of the NC emissions. The second group of emissions is presented in Table 3 and is related to the NCs (centered around λ_em_ = 580–660 nm depending on the type of NC). This does not mean that these emission processes come directly from the metal cores; instead, they come from ligand-to-metal charge transfer processes (LMCT), as it is supported by the µs lifetimes [26]. The largest component varies greatly in value (1.21–3.59 µs).

There are two more components, one with 86.2–503.9 ns lifetime, and another with 2.54–31.3 ns value, the latter being unreliable, as supported by the large 3σ values. The presence of NC-related emissions is hard to detect due to their usually low QY, but in their case, lifetimes should be in the ns region [27]. The LMCT processes also explain the high QY values obtained for protein-stabilized NCs. As was explained in the previous section, the emission maxima related to NCs can be deconvoluted into three major components (noted with arrows 1–3 in Figure 2). These components have their maxima at 660 nm, 570 nm, and 610 nm in order of increasing silver content. The first one is clearly related to Au NCs, while the latter ones are bimetallic NCs with lower and higher Ag contents. Further emission peaks were not identified, suggesting that higher silver contents either disrupt the emission process, or the changes in core structure create non-radiative species. This is somewhat supported by the fact that pure Ag NCs made with our preparation methods were not fluorescent (γG [17]), or had very weak fluorescence at λ_em_ = 730 nm [15]. NCs made with LYZ, using an additional reducing agent (dithiothreitol [28]) responsible for sulfur bond breaking, produce NCs with an emission centered at λ_em_ = 640 nm; as such, the emission in bimetallic NCs cannot be the simple mixture of Au and Ag NC emissions. On a side note, disulfide bond number in the used proteins did not have any effect on NC emission (both intensity and peak position). The numbers of these structure-determining elements are as follows: 17 in BSA, 4 in LYZ, 16–25 in γG (depending on immunoglobulin type [29]), 19 in Tf and 17 in HSA.

The optical behavior of NCs can also be investigated using fluorescence quenchers such as iodide (I^−^) or acrylamide (AAm). These molecules can quench the fluorescence of tryptophan, giving information about the position of these residues in the structure of the stabilizing proteins. The experiments using these materials were carried out in three different media, namely 0.1 M HCl, Na_2_HPO_4_ and NaOH, to check whether different pHs can alter protein structure significantly. Based on our results, protein type combined with the appropriate quencher gave mixed results. General changes are marked in Table S1. I^−^ could quench the fluorescence of BSA-, HSA- and LYZ I-Au/Ag NCs in acidic medium, while an increase happened with γG-Au/Ag NCs. The LYZ II-Au/Ag NCs did not show any obvious change. For AAm quenching, instead, emission enhancement happened for BSA-, LYZ I- and γG-Au/Ag NCs in both acidic media and Na_2_HPO_4_. LYZ II-Au/Ag NCs showed decline (quenching) in Na_2_HPO_4_, and mixed results in acidic medium. The cases where quenching happened could not be elaborated further, as neither wavelength shift nor peak shape distortion could be observed, unlike in the case of Tf-Au/Ag NCs in a previous work [16].

Assessing the performance of fluorescent NCs as emitters can be done by measuring their QY. Such measurements were done previously by our group [15,16,17], and new measurements were executed on the freshly prepared HSA-Au/Ag NCs (at λ_ex_ = 325 and 365 nm). As Table 4 shows, bimetallic NCs generally have higher QY compared to their monometallic counterparts (Au NCs, see references [15,16,17]). HSA-Au/Ag NCs possess similar QY as other bimetallic NCs, but much lower QY than LYZ-based ones.

### 3.4. Structural Assessment

In a general sense, the investigation of protein-stabilized NCs involves two major targets: the metal core and the protein, and additionally, the whole structure is studied (e.g., size and stability and optical properties). The first major information we obtained is the actual metal content of the synthesized NCs. For this reason, ICP-MS measurements were conducted on every sample, and the results are presented in Table 5.

**Figure 3:**
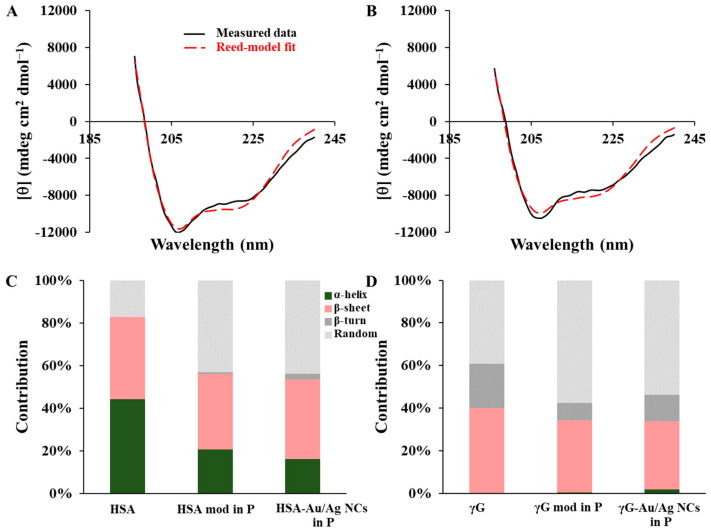
The CD spectrum of NaOH-treated HSA (HSA mod, (**A**)) and HSA-Au/Ag NCs (**B**) in 10 mM Na_2_HPO_4_ solution (P). (**C**,**D**) The calculated secondary structure of different HSA and γG forms (pure, treated and NC) in P solution, based on the Reed model.

As can be seen, the cleaning process applied to every NC decreases the metal content of the product, which is more noticeable in the case of gold. However, silver content usually remained over 75%, while in the case of Tf-Au/Ag NCs, higher than 100% was measured, which can be explained by failed preparation and errors coming from dilution. In the next set of measurements, CD data were collected to check whether NC formation causes the changes in protein structure, or whether the denaturation is simply caused by the high-pH environment. For this reason, a parallel set of samples were made, where pure proteins were also prepared using the same conditions as for NCs in Section 2.2. In this section, our results were compared to already published results of the pristine proteins [15,16,17]. For the evaluation of data, two different estimation models, namely the Yang and Reed models, were used. The former is preferentially used for larger proteins, while the latter is used for smaller oligopeptides. In this case, the Yang model usually provided a slightly better fit compared to the Reed model for the unmodified protein samples. On the other hand, the NC samples could not be fitted properly with this model; thus, the Reed model was used in all cases for comparison purposes (see Figure 3A,B). Based on our results, NC formation has less effect compared to NaOH. In high-pH conditions, the protein molecule unfolds, losing much of its ordered structure, especially α-helices (in the case of γG, where no α-helices are present, the contribution of β-turns becomes lower; see Figure 3C,D).

Major differences come from the protein itself. The secondary structure of the investigated proteins is well-known in the literature. HSA mainly has α-helices (67%) as structure-determining elements, and in solvated form, the random parts and β-turns are also prominent [30]. β-sheets are lacking in the pristine protein, but they can appear when the protein becomes denatured. In the presented Reed model-based structure compositions, β-sheets did appear as main elements, showing that this model misjudges the composition of the protein. However, in the case of treated HSA and HSA-Au/Ag NCs, this model is appropriate, as denaturation caused by NaOH and NC formation can make these secondary structure elements appear. If we compare the pristine proteins, the pH-treated ones, and the NCs, we can clearly see that the latter two structures are more similar, and this proves that NC formation itself does not cause major changes.

### 3.5. XPS Studies

**Figure 4:**
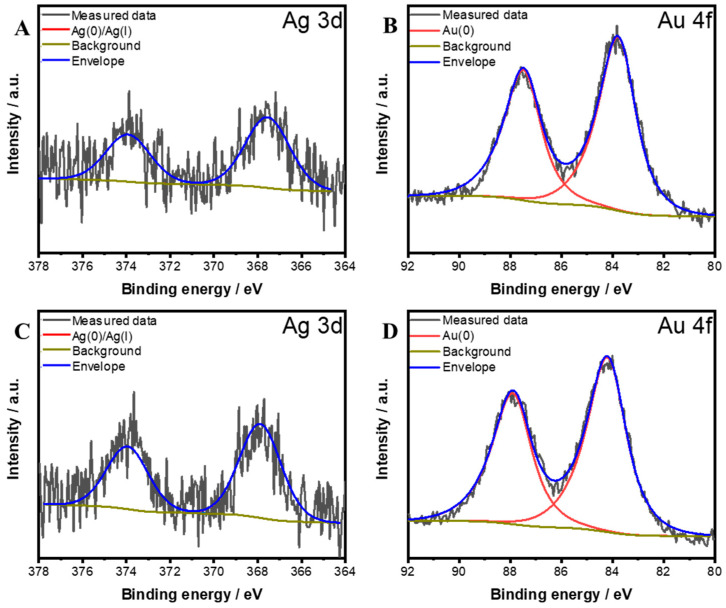
Core-level X-ray photoelectron spectrum of HSA-Au/Ag NCs (**A**,**B**) and Tf-Au/Ag NCs (**C**,**D**). (**A**,**C**) Ag 3d peaks; (**B**,**D**) Au 4f peaks.

XPS studies conducted on the NCs gave insights on the surface elemental composition and oxidation state. In our case, the silver content of optimized Au/Ag NCs was too low to measure (see references [15,17]), so these experiments were conducted on samples with higher metal contents. Even with this change, the metal content remained very low (Au at ~1 at% out of all elements). It is obvious that with this method, information on the optimized NCs could not be collected, and the results from the NCs with higher metal content will slightly differ from the actual state of the optimized NCs. For every sample, survey scans were acquired (these are shown in Figure S4), and the major elements that could be detected were C, N, Au and Ag. The C 1s and N 1s spectra (presented in Figures S5 and S6) showed almost identical features, where the complexity of the proteins inhibited the separation of different chemical environments. As is presented in Figure 4 for HSA-Au/Ag NCs and Tf-Au/Ag NCs and Figure S7 for other protein-stabilized NCs, the presence of silver could be detected, although LYZ II-Au/Ag NCs had very low amounts, on the edge of (rather below) the device’s measurement limits (see Figure S7C). As such, the quantitative data arising from silver content should be treated cautiously, as the signal to noise ratio was very low in every case.

Nevertheless, from the results we could see that Ag showed a peak at 367.4–367.9 eV (see Table 6), which is somewhat different from metallic Ag^0^ 3d_3/2_ and Ag^0^ 3d_5/2_ peaks (positioned at 374.3 and 368.2 eV, respectively [31,32]) in some cases. A common identifier of Ag^0^ state is the difference of ~6.0 eV between the Ag 3d_3/2_ and Ag 3d_5/2_ peaks [31,33], which is clearly true for BSA-, LYZ I-, LYZ II- and Tf-Au/Ag NCs (differences of 5.9–6.1 eV), and is within signal noise for HSA-Au/Ag NCs (6.3 eV). In the case of γG-Au/Ag NCs, the Ag^0^ 3d_3/2_ peak is situated at higher energies, which might be due to low signal. Overall, the peak positions are somewhat lower than pure Ag^0^ would suggest, where different reasons could play a role: (i) the shift could be due to the presence of surface Ag(I) species, or (ii) from the electronic effect of Au-Ag alloy formation. The shift in Ag 3d peaks is cited in many articles [33,34,35], but without any further explanation. The thumb rule of oxidation states in XPS would suggest that Ag(I) species should appear at higher binding energies (such as Ag^+^ in AgCl at 368.1 or 368.9 eV [31,36] or in Ag_2_O at 368.2–368.4 eV [31]). From the former studies and handbooks available for XPS data [33,34,35,37,38,39], we could see that the position of Ag 3d peaks can be affected by various effects; thus, the clear separation of Ag^0^ and Ag(I) peak positions was not possible. The peak related to gold is present at 83.7–84.1 eV (see Figure 4B,D, and Figure S8).

Compared with earlier results, Au is mainly present in metallic (Au^0^) form, but this value can also suggest the presence of a small amount of Au(I) species. The presence of Au(I) species explains the high (µs range) photoluminescence lifetimes and LMCT processes involving Au(I) and sulfur atoms. The shift to lower energies for both Au and Ag (compared to bulk metals) can be explained by particle size-related effects [40], more exactly initial and final state effects, which are more significant at very low sizes (in this case the sizes were around 2–3 nm; see references [15,16,17]). The composition of metals showed higher Ag content than the nominal value (see Table 6), which is in accordance with the results of ICP-MS measurements. As XPS is a surface-sensitive technique, the results could also suggest that silver might be accumulated on the surface of the bimetallic NCs, but due to low signals, these calculations can be misleading. The possible presence of Ag(I) in the surface region of NCs can also contribute to LMCT processes, explaining the shift in λ_em_ values and the changes in emission intensities. The most important result from these measurements is that at the end of NC formation, most of the Au species are zero valent, but some Au(I) also remains (or is subsequently formed during cleaning or storage). In the case of silver, the exact ratio of Ag^0^ to Ag(I) cannot be determined, but the electrochemical properties of Ag suggest higher amounts of Ag(I) compared to the less reactive gold.

### 3.6. Investigation of Biocompatibility

The cytotoxic effect and antibacterial activity of some of the prepared NCs indicated in Table 7 and Table 8 were tested by determining the half maximal inhibitory concentrations (IC_50_) and the minimum inhibitory concentration (MIC). The selection was based on fluorescence; formulations with emissions in the so-called biological window (λ_em_ > 600 nm) were chosen, and from the serum-albumin-based samples, only HSA-Au NCs and Au/Ag NCs were tested. The DOX used as reference in cytotoxicity assays showed an IC_50_ of 0.71 µM for Colo205 and 0.26 µM for CCD-19Lu cells. Compared to this, as our results show in Table 7 and Table 8, no NCs showed any effect in both tests, meaning that the formed products did not show any cytotoxic effect on the tested cells, and also no growth inhibition up to 50 µM was detected on the tested bacterial strains. The only exceptions were Tf-Au/Ag NCs that showed cytotoxic activity at very high concentrations (IC_50_ of >25 µM). This effect was due to the low concentration of NCs in the sample, which required a large amount to be added to the sample well plates, and this immensely diluted the culture liquid, indirectly decreasing the viability of the tested cells by inducing osmotic stress or nutrient depletion.

## 4. Conclusions

In this study, we successfully developed a newly optimized, template-assisted fabrication route at 25 °C to synthesize highly fluorescent HSA-stabilized bimetallic Au/Ag NCs. By comparing these with newly prepared and previously reported NCs stabilized by BSA, LYZ, Tf, and γG, we systematically demonstrated that the choice of protein template and the precise control of metal stoichiometric ratios are the primary determinants of the resulting bimetallic cores and their emission properties. Specifically, three distinct emission pathways (at 660 nm, 570 nm, and 610 nm) were identified as a function of Ag incorporation, governed by LMCT dynamics. Structural and surface analyses via CD and XPS confirmed that the initial high-pH conditions (rather than the NC formation itself) induce partial protein unfolding while maintaining the zero-valent dominance of the core metals. Crucially, in vitro biological assays verified that these bimetallic NCs exhibit excellent biocompatibility, possessing no inherent cytotoxicity toward human cancer and normal fibroblast lines nor any significant antibacterial activity against the tested Gram-positive and Gram-negative strains within the tested concentration range and exposure conditions.

Despite these promising features, some limitations of the current study should be noted. First, the necessity of utilizing basic conditions (high pH) to initiate the protein-mediated reduction restricts the preservation of the fully native state of the protein templates. Second, while the purification via dialysis maintains colloidal stability, it inevitably leads to a slight loss of the initial metal precursors (particularly gold). Furthermore, although XPS measurements confirmed alloy/core characteristics, the exceptionally low metal-to-protein ratio in the optimized structures poses challenges for atomic-level surface resolution without synthesizing dedicated high-metal-content samples.

Nevertheless, the non-toxic nature, tunable photophysical profiles, and microsecond-range fluorescence lifetimes of these biocompatible bimetallic NCs make them highly qualified candidates for advanced biophotonics. In particular, their long lifetimes are highly promising for time-resolved fluorescence imaging to suppress biological background autofluorescence, while the preserved structural loops of specific proteins (such as transferrin or immunoglobulins) open up strategic pathways for targeted drug delivery, active biosensing, and in vivo diagnostic imaging.

## Figures and Tables

**Table 1:** The composition of the Au/Ag bimetallic NCs stabilized by various proteins based on Refs. [15,16,17], and the composition of the NC samples with increased metal content prepared only for XPS studies (In the case of LYZ, we prepared samples with two compositions, LYZ-I and LYZ-II).

Sample	V_sample_ (mL)	Optimized NCs			NCs for XPS		
		m_protein_ (mg)	m_Au_ (mg)	m_Ag_ (µg)	m_protein_ (mg)	m_Au_ (mg)	m_Ag_ (µg)
BSA-Au/Ag NCs	5.98	45	0.453	27.0	15	0.906	53.9
LYZ I-Au/Ag NCs	5.98	45	0.428	40.5	20	0.857	80.9
LYZ II-Au/Ag NCs	5.88	60	0.355	12.2	20	0.945	32.4
γG-Au/Ag NCs	5.91	90	0.344	18.3	15	0.916	48.5
Tf-Au/Ag NCs	4.60	5.4	0.071	4.5	3.0	0.282	18.1
HSA-Au/Ag NCs	5.88	45	0.680	40.5	15	0.906	53.9

**Table 2:** The lifetime components of different protein-stabilized Au/Ag NCs at λ_em_ = 460 nm (λ_ex_ = 371 nm).

4-Parameter Fit	BSA	3σ	HSA	3σ	LYZ I	3σ	LYZ II	3σ	γG	3σ	Tf	3σ
**λ_em_ (nm)**	460		460		460		460		460		460	
**Frequency (MHz)**	8		8		4		4		8		4	
**Time range (fitted) (ns)**	85		90		150		200		85		120	
**Max counts**	10,000		10,000		50,000		50,000		10,000		10,000	
**τ_1_ (ns)**	0.185	0.047	0.208	0.020	0.265	0.029	0.311	0.033	0.254	0.051	0.268	0.068
**τ_2_ (ns)**	1.04	0.10	0.99	0.23	1.42	0.08	1.60	0.08	1.20	0.12	1.11	0.16
**τ_3_ (ns)**	3.59	0.08	3.25	0.19	4.15	0.11	4.68	0.06	3.84	0.20	3.47	0.26
**τ_4_ (ns)**	9.39	0.61	9.31	0.26	11.5	0.3	13.4	0.8	9.95	0.49	9.43	0.45
**A_1_ (%)**	7.17		11.66		14.77		12.71		9.71		13.05	
**A_2_ (%)**	21.87		19.71		31.08		34.33		26.23		27.35	
**A_3_ (%)**	49.58		44.17		44.15		45.94		48.93		42.96	
**A_4_ (%)**	21.38		24.45		9.99		7.02		15.13		16.64	
**χ^2^**	1.042		1.217		1.222		1.340		1.083		1.201	

**Table 3:** The lifetime estimations of different protein-stabilized Au/Ag NCs at the NC-related λ_em_ values (λ_ex_ = 371 nm).

3-Parameter Fit	BSA	3σ	HSA	3σ	LYZ I	3σ	LYZ II	3σ	γG	3σ	Tf	3σ
**λ_em_ (nm)**	640		640		620		580		660		660	
**Frequency (kHz)**	50		100		50		100		50		50	
**Time range (fitted) (µs)**	10		6.8		10		6.8		8		7	
**Max counts**	300		200		500		2000		300		300	
**τ_1_ (ns)**	31.3	29.4	2.54	2.07	9.82	0.44	2.86	0.27	36.8	35.9	7.32	2.41
**τ_2_ (ns)**	657.2	73.9	86.2	42.9	360.3	50.2	278.1	62.8	503.9	157.2	397.2	246.3
**τ_3_ (μs)**	3.59	0.32	1.34	0.07	2.00	0.08	1.21	0.07	1.75	0.10	2.49	0.39
**A_1_ (%)**	0.88		1.06		0.9		8.87		0.74		1.73	
**A_2_ (%)**	19.06		3.68		7.73		23.12		15.41		8.98	
**A_3_ (%)**	80.06		95.26		91.37		68.01		83.85		89.29	
**χ^2^**	1.671		1.342		1.303		1.236		1.013		1.086	

**Table 4:** The characteristic excitation (λ_ex_) and emission (λ_em_) as well as the internal QY values with the corresponding standard deviations (SDs (%)) of different protein-stabilized bimetallic NCs (number of technical replicates was 3).

Sample	λ_ex_ (nm)	λ_em_ (nm)	QY (%)	SD (%)
BSA-Au/Ag NCs	365	622	2.47 [15]	0.61
LYZ I-Au/Ag NCs	370	603	7.44 [15]	0.59
LYZ II-Au/Ag NCs	370	563	4.78 [15]	0.34
γG-Au/Ag NCs	365	637	2.31 [17]	0.29
Tf-Au/Ag NCs	365	613	3.46 [16]	0.30
HSA-Au/Ag NCs	325	612	3.28	0.59
	365	612	3.56	0.37

**Table 5:** The nominal and actual metal composition of different NCs after cleaning based on ICP-MS data (RSD of individual measurements was between 5 and 10%). The results of BSA-, LYZ I-, LYZ II-, γG and Tf-Au/Ag NCs originate from references [15,16,17].

Samples	*ρ*_nominal_ (µg mL^−1^)		*ρ*_measured_ (µg mL^−1^)		% of Metal Remaining	
	Au	Ag	Au	Ag	Au	Ag
BSA-Au/Ag NCs	75.75	4.52	52.50	3.88	69	86
LYZ I-Au/Ag NCs	71.64	6.76	40.87	5.17	57	76
LYZ II-Au/Ag NCs	60.30	2.07	39.44	1.62	65	78
γG-Au/Ag NCs	58.16	3.10	43.50	2.90	75	93
Tf-Au/Ag NCs	15.30	0.99	11.92	1.16	78	117
HSA-Au/Ag NCs	75.75	4.51	45.71	4.27	60	95

**Table 6:** The summary of Au 4f and Ag 3d peak positions, as well as estimated atomic percentage (at%) of the metals in the bimetallic NCs.

Sample	Au				Ag			
	4f_5/2_ (eV)	4f_7/2_ (eV)	Calculated at%	Nominal at%	3d_3/2_ (eV)	3d_5/2_ (eV)	Calculated at%	Nominal at%
BSA-Au/Ag NCs	87.6	83.9	86	90	373.7	367.8	14	10
LYZ I-Au/Ag NCs	87.4	83.7	84	85	373.5	367.4	16	15
LYZ II-Au/Ag NCs	87.4	83.7	80	94	373.3	367.4	20	6
γG-Au/Ag NCs	87.9	84.2	84	91	374.7	367.9	16	9
Tf-Au/Ag NCs	87.9	84.2	85	90	374.0	367.9	15	10
HSA-Au/Ag NCs	87.5	83.8	85	90	373.9	367.6	15	10

**Table 7:** The cytotoxic effect of the investigated gold and bimetallic NCs on Colo205 and CCD-19Lu cells, based on MTT-assay. The IC_50_ values are given in µM, representing the total metal concentration of NCs.

Cytotoxic Effect				
Samples	Colo205 Cells		CCD-19Lu Cells	
	IC_50_ (µM)	SD +/−	IC_50_ (µM)	SD +/−
DOX	0.71	0.01	0.26	0.16
LYZ I-Au NCs	>50	-	>50	-
γG-Au NCs	>50	-	>50	-
Tf-Au NCs	>50	-	>50	-
HSA-Au NCs	>50	-	>50	-
LYZ I-Au/Ag NCs	>50	-	>50	-
γG-Au/Ag NCs	>50	-	>50	-
Tf-Au/Ag NCs	>25	-	>25	-
HSA-Au/Ag NCs	>50	-	>50	-

**Table 8:** The antibacterial effect of the investigated NCs on four strains of bacteria, based on MIC-determination method. The MIC values are given in µM, representing the total metal concentration of NCs.

MIC Determination (µM)				
Samples	*S. aureus*	*S. aureus* (MRSA)	*E. coli*	*K. quasipneumoniae*
	ATCC 25923	ATCC 43300	ATCC 25922	ATCC 700603
LYZ I-Au NCs	>50	>50	>50	>50
γG-Au NCs	>50	>50	>50	>50
Tf-Au NCs	>50	>50	>50	>50
HSA-Au NCs	>50	>50	>50	>50
LYZ I-Au/Ag NCs	>50	>50	>50	>50
γG-Au/Ag NCs	>50	>50	>50	>50
Tf-Au/Ag NCs	>50	>50	>50	>50
HSA-Au/Ag NCs	>50	>50	>50	>50

## Data Availability

The original contributions presented in this study are included in the article/Supplementary Material. Further inquiries can be directed to the corresponding author.

## Supplementary Materials

The following supporting information can be downloaded at https://www.mdpi.com/article/10.3390/nano16150909/s1, Figure S1. (A) Effect of synthesis temperature on NC emission profiles. (B) The effect of total metal concentration (c_Me_, mM) on the normalized emission intensity (I_norm_, cps/mM) of the bimetallic NCs (measured at λ_max_); number of replicates was n = 3; Figure S2. (A) The evolution of NC emission spectrum based on synthesis time. (B) The change in emission intensities at λ = 440 nm (protein, ●) and at λ = 555–616 nm (NC emission, ♦). The number of replicate measurements was n = 3; Figure S3. The absorbance (Abs), excitation (Exc) and emission spectra (Em) of HSA-Au NCs (A–C) and HSA-Au/Ag NCs (n_Au_/n_Ag_ = 45:1) with 1×, 10× and 100× dilution (D–F); Figure S4. The survey scans of protein-stabilized bimetallic NCs: (A) BSA-Au/Ag NCs, (B) LYZ I-Au/Ag NCs, (C) LYZ II-Au/Ag NCs, (D) γG-Au/Ag NCs, (E) HSA-Au/Ag NCs, (F) Tf-Au/Ag NCs; Figure S5. The C 1s spectra of the investigated NC-samples: (A) BSA-Au/Ag NCs, (B) LYZ I-Au/Ag NCs, (C) LYZ II-Au/Ag NCs, (D) γG-Au/Ag NCs, (E) HSA-Au/Ag NCs, (F) Tf-Au/Ag NCs; Figure S6. The N 1s spectra of the bimetallic NCs: (A) BSA-Au/Ag NCs, (B) LYZ I-Au/Ag NCs, (C) LYZ II-Au/Ag NCs, (D) γG-Au/Ag NCs, (E) HSA-Au/Ag NCs, (F) Tf-Au/Ag NCs; Figure S7. The Ag 3d spectra of protein-stabilized bimetallic NCs: (A) BSA-Au/Ag NCs, (B) LYZ I-Au/Ag NCs, (C) LYZ II-Au/Ag NCs, (D) γG-Au/Ag NCs; Figure S8. The Au 4f spectra of four protein-stabilized bimetallic NCs: (A) BSA-Au/Ag NCs, (B) LYZ I-Au/Ag NCs, (C) LYZ II-Au/Ag NCs, (D) γG-Au/Ag NCs; Table S1. The effect of quenchers iodide (I^−^) and acrylamide (AAm) on the emission intensities of different protein-stabilized Au/Ag NCs.
