# Supplementary material for: Protein-Mediated Bimetallic Nanoclusters: Effect of Protein Nature on Structure, Optical Property and Cytotoxicity
Source: Nanomaterials (Basel). 2026 Jul 24;16(15):909. doi: 10.3390/nano16150909 (PMC13468674; doi:10.3390/nano16150909)
Supplement: Supplementary file 1 [file nanomaterials-16-00909-s001.zip › nanomaterials-4444465-supplementary.pdf]

nanomaterials

Supporting information

Protein-mediated bimetallic nanoclusters: effect of protein nature on structure, optical property and cytotoxicity

Bianka Torma [1], Gergely F. Samu [2], Gabriella Spengler [3], Edit Csapó [1,*] and Árpád Turcsányi [1,*]

[1] MTA-SZTE Lendület "Momentum" Noble Metal Nanostructures Research Group, Department of Physical Chemistry and Materials Science, University of Szeged, Rerrich B. Square 1, H-6720 Szeged, Hungary; torma.bianka@chem.u-szeged.hu
[2] Department of Molecular and Analytical Chemistry, University of Szeged, Dóm Square 7–8, H-6720 Szeged, Hungary; samugf@chem.u-szeged.hu
[3] Department of Medical Microbiology, Albert Szent-Györgyi Medical School, University of Szeged, Semmelweis utca 6, H-6725 Szeged, Hungary; spengler.gabriella@med.u-szeged.hu
* Correspondence: juhaszne.csapo.edit@med.u-szeged.hu (E.C.); tarpad@chem.u-szeged.hu (Á.T.)

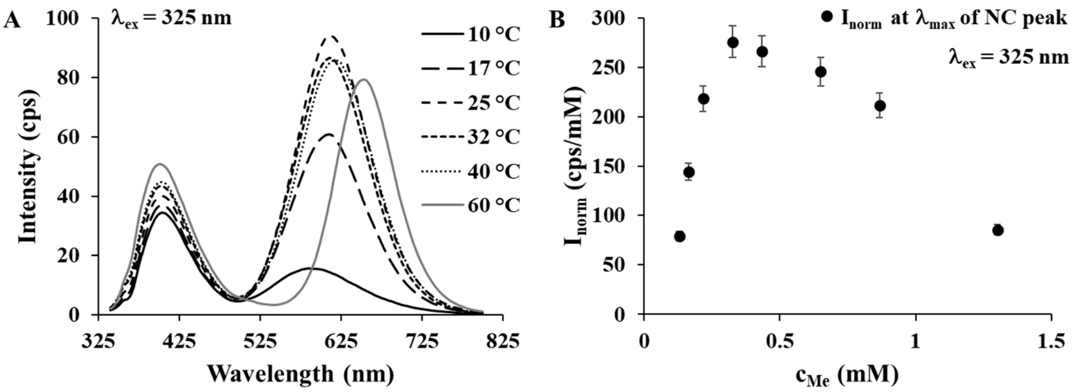

Figure S1. (A) Effect of synthesis temperature on NC-emission profiles **(B)** The effect of total metal concentration (c_{Me}, mM) on the normalized emission intensity (I_{norm}, cps/mM) of the bimetallic NCs (measured at λ_{max}; number of replicates was n = 3).

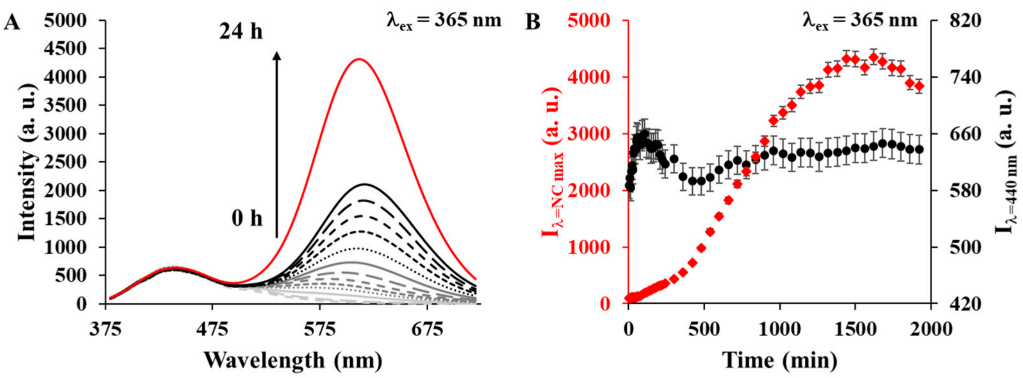

Figure S2. (**A**) The evolution of NC-emission spectrum based on synthesis time **(B)** The change in emission intensities at λ = 440 nm (protein, ●) and at λ = 555–616 nm (NC-emission, ♦). The number of replicate measurements was n = 3.

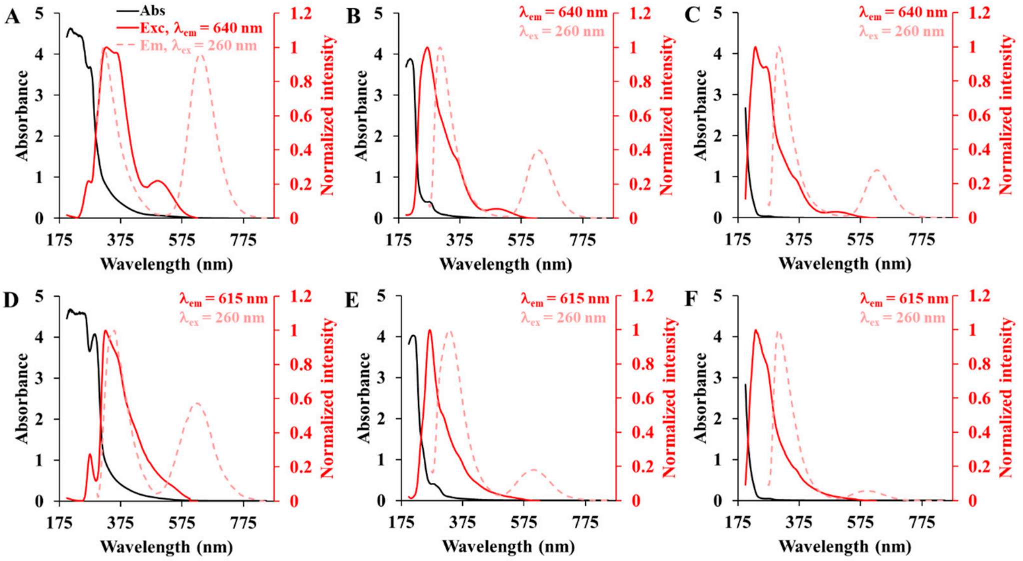

Figure S3. The absorbance (Abs), excitation (Exc) and emission spectra (Em) of HSA-Au NCs (**A**–**C**) and HSA-Au/Ag NCs (n_{Au}/n_{Ag} = 45:1) with 1×, 10× and 100× dilution (**D**–**F**).

Table S1. The effect of quenchers iodide (I^-) and acrylamide (AAm) on the emission intensities of different protein-stabilized Au/Ag NCs.

Effect of I^-/Aam	Medium		
	**0.1 M HCl**	**0.1 M NaOH**	**0.1 M Na_2HPO_4**
BSA-Au/Ag NCs	↓/n.a. (↑)	n.a./↑	n.a./↑
LYZ I-Au/Ag NCs	↓/↑	n.a./n.a.(↑)	–/–
LYZ II-Au/Ag NCs	n.a./n.a.	n.a./↓	–/–
γG-Au/Ag NCs	↑/↑	↓/n.a.	n.a./↑
HSA-Au/Ag NCs	↓/–	n.a./–	n.a./–

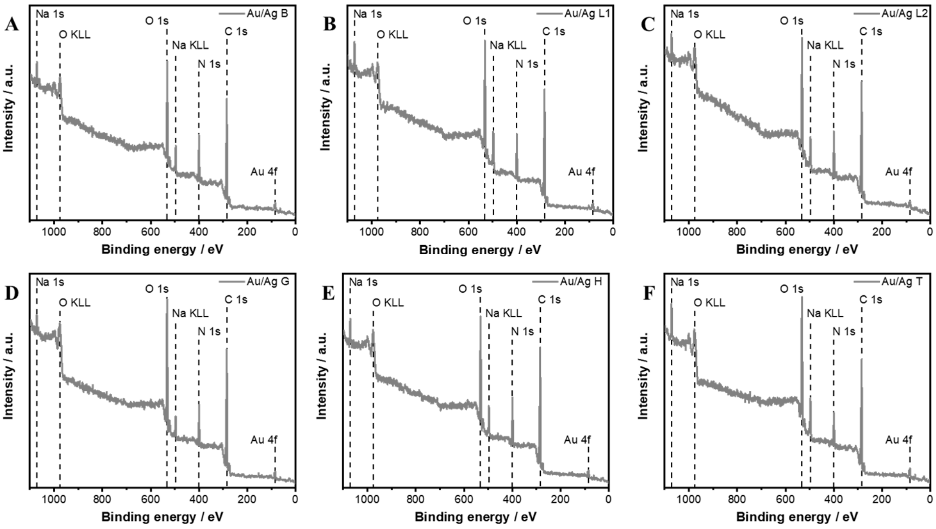

Figure S4. The survey scans of protein-stabilized bimetallic NCs **(A)** BSA-Au/Ag NCs **(B)** LYZ I-Au/Ag NCs **(C)** LYZ II-Au/Ag NCs **(D)** γG-Au/Ag NCs **(E)** HSA-Au/Ag NCs **(F)** Tf-Au/Ag NCs.

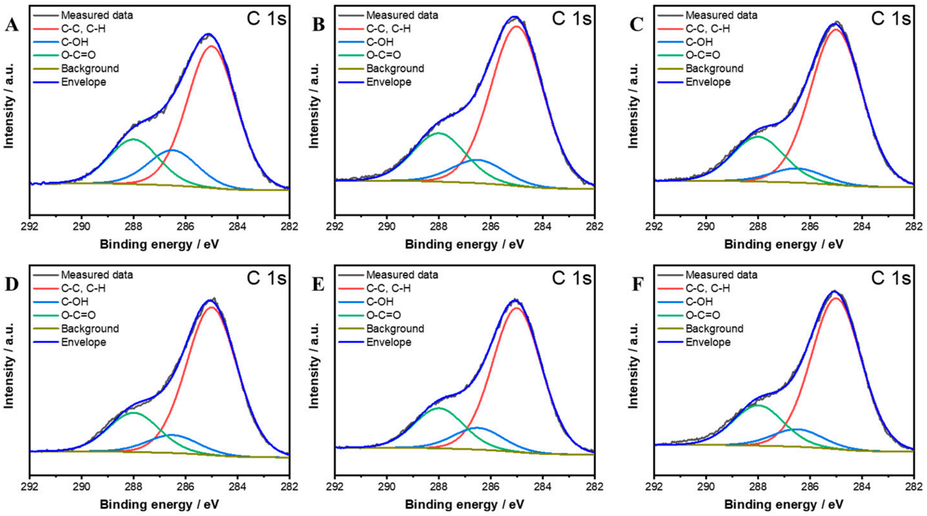

Figure S5. The C 1s spectra of the investigated NC-samples **(A)** BSA-Au/Ag NCs **(B)** LYZ I-Au/Ag NCs **(C)** LYZ II-Au/Ag NCs **(D)** γG-Au/Ag NCs **(E)** HSA-Au/Ag NCs **(F)** Tf-Au/Ag NCs.

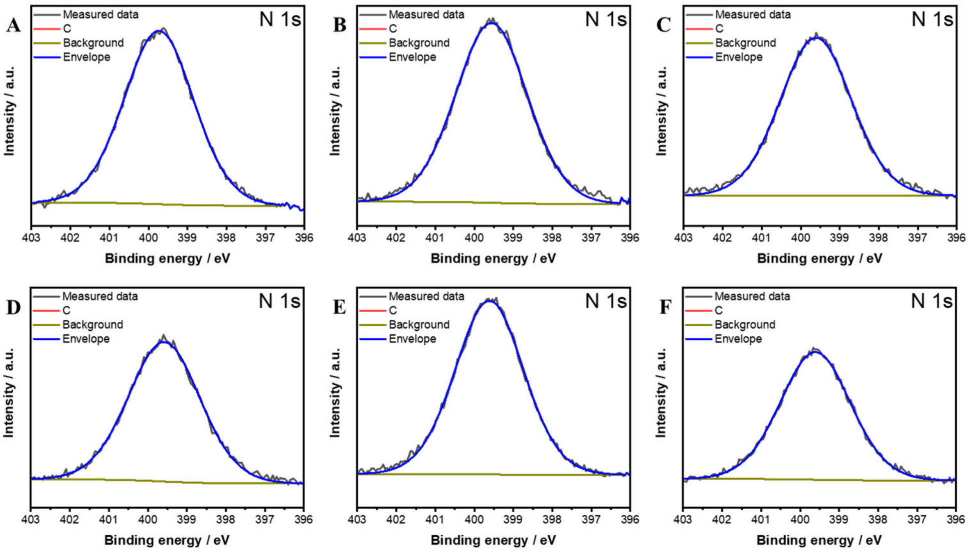

Figure S6. The N 1s spectra of the bimetallic NCs **(A)** BSA-Au/Ag NCs **(B)** LYZ I-Au/Ag NCs **(C)** LYZ II-Au/Ag NCs **(D)** γG-Au/Ag NCs **(E)** HSA-Au/Ag NCs **(F)** Tf-Au/Ag NCs.

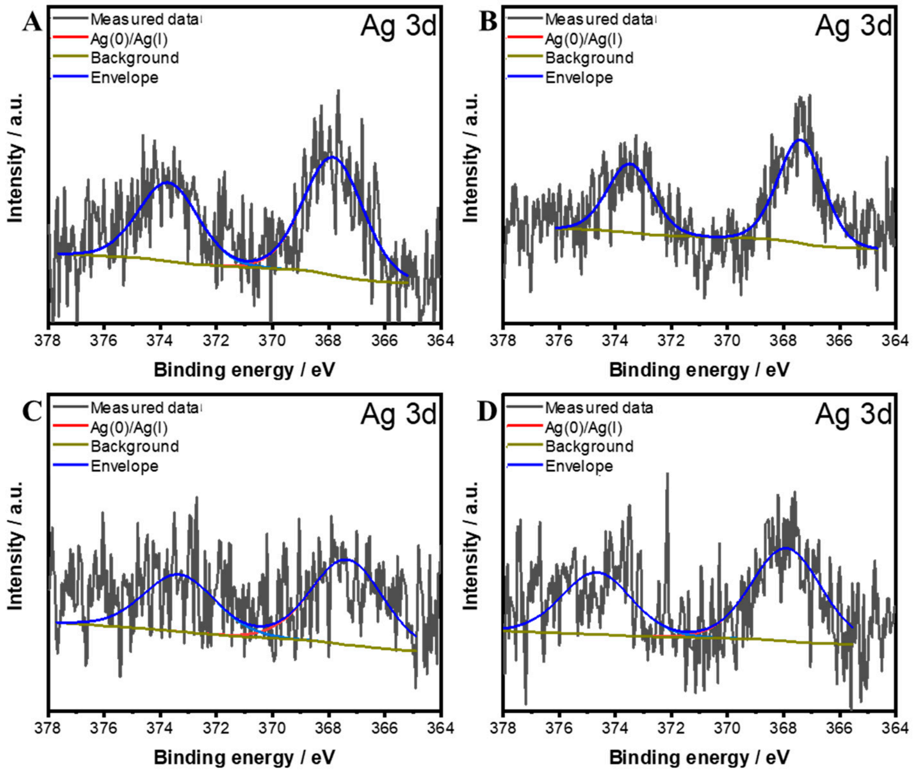

Figure S7. The Ag 3d spectra of protein-stabilized bimetallic NCs **(A)** BSA-Au/Ag NCs **(B)** LYZ I-Au/Ag NCs **(C)** LYZ II-Au/Ag NCs **(D)** γG-Au/Ag NCs.

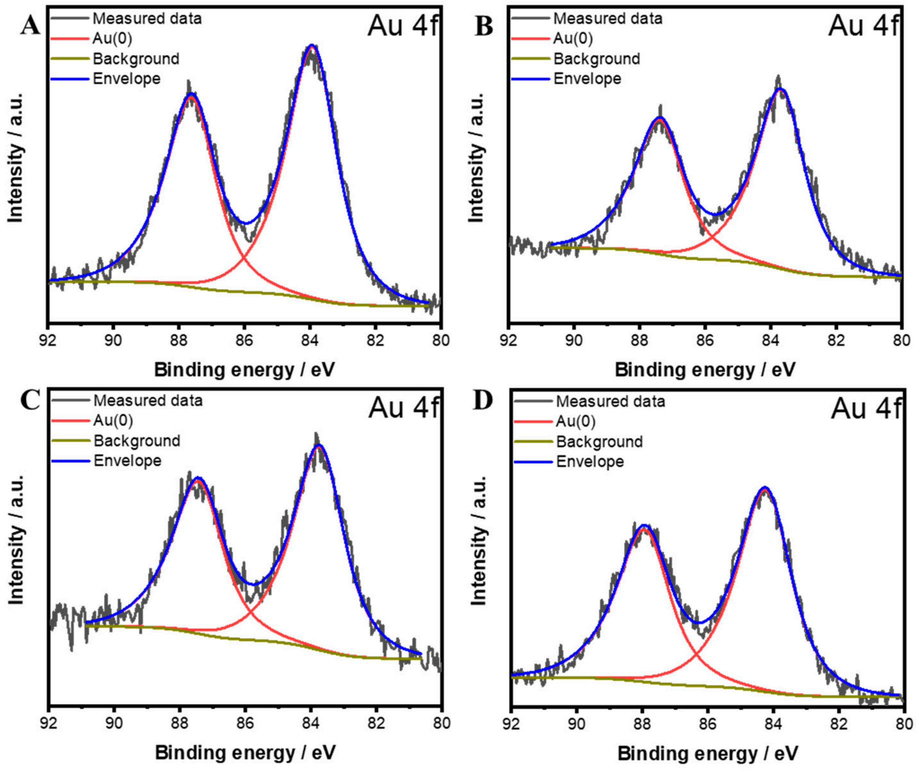

Figure S8. The Au 4f spectra of four protein-stabilized bimetallic NCs **(A)** BSA-Au/Ag NCs **(B)** LYZ I-Au/Ag NCs **(C)** LYZ II-Au/Ag NCs **(D)** γG-Au/Ag NCs.
